# Supplementary material for: Fission yeast Srr1 and Skb1 promote isochromosome formation at the centromere
Source: Commun Biol. 2023 May 26;6:551. doi: 10.1038/s42003-023-04925-9 (PMC10219947; doi:10.1038/s42003-023-04925-9)
Supplement: Supplementary file 2 — Supplementary Information [file 42003_2023_4925_MOESM2_ESM.pdf]

Supplementary Information

Supplementary Figures 1-6
Supplementary Tables 1-2

Fission yeast Srr1 and Skb1 promote isochromosome formation at the centromere

Piyusha Mongia, Naoko Toyofuku, Ziyi Pan, Ran Xu, Yakumo Kinoshita,
Keitaro Oki, Hiroki Takahashi, Yoshitoshi Ogura, Tetsuya Hayashi, Takuro Nakagawa

Supplementary Fig. 1

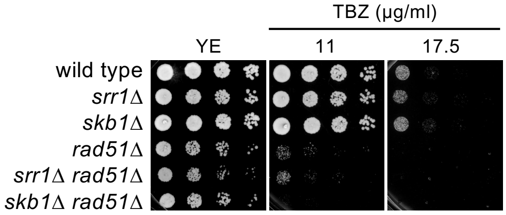

Supplementary Fig. 1 A serial dilution assay to determine the TBZ sensitivity of wild-type, *srr1*Δ*, skb1*Δ, *rad51*Δ*, srr1*Δ *rad51*Δ*,* and *skb1*Δ *rad51*Δ strains (TNF35, 5943, 8321, 8107, 8188, and 8320). Exponentially growing cells were spotted onto YE plates supplemented with the indicated concentrations of TBZ.

Supplementary Fig. 2

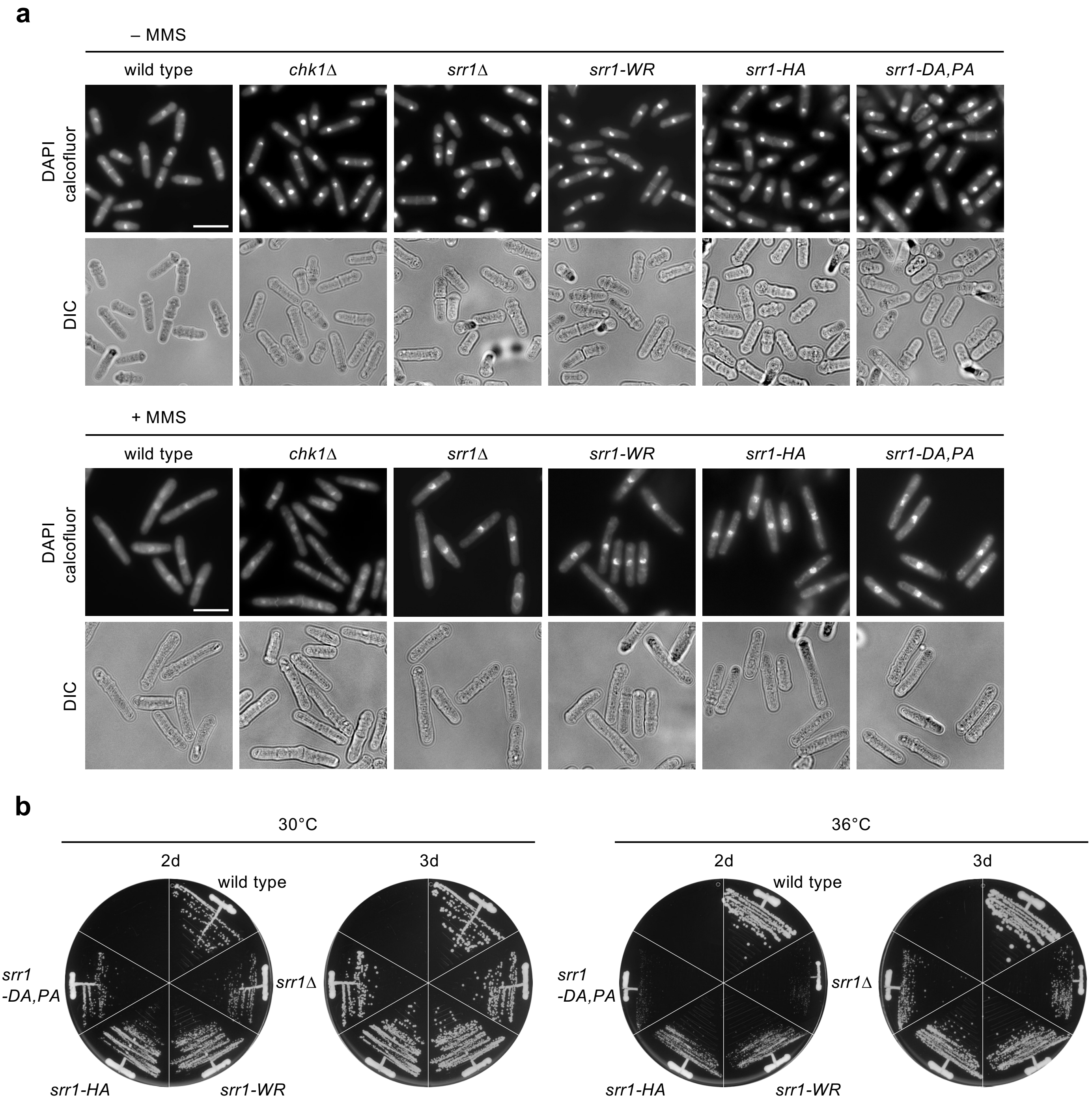

Supplementary Fig. 2 a Cell morphology of wild-type, *chk1*Δ, *srr1*Δ, *srr1-W157R*, *srr1-H148A*, and *srr1-D111A,P112A* strains (TNF35, 3559, 5943, 9011, 9007, and 8990). Early log-phase EMM cultures were divided into two aliquots and incubated for 8 h at 30ºC either in the presence (bottom row) or absence (top row) of 0.01% MMS. DIC, differential interference contrast. A bar indicates 10 µm. Cells were observed using a DeltaVision Personal fluorescence microscopy system (see Methods). **b** Colony formation of wild-type, *srr1*Δ, *srr1-W157R*, *srr1-H148A*, and *srr1-D111A,P112A* strains (TNF3885, 5847, 8280, 8275, and 8274) on YE3S plates. Note that, compared to wild-type cells, *srr1*Δ, *srr1-W157R*, *srr1-H148A*, and *srr1-D111A,P112A* strains formed small colonies, especially at 36ºC.

Supplementary Fig. 3

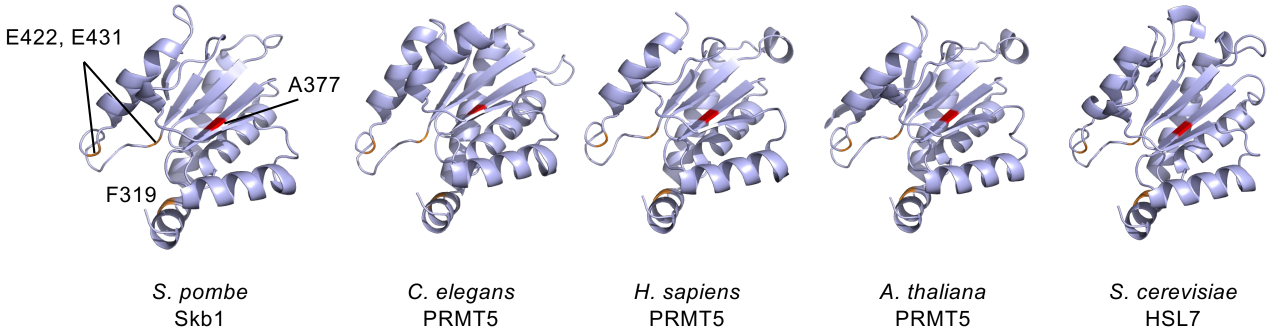

Supplementary Fig. 3 The RMTsae domain structure of *S. pombe* Skb1 and the homologs are shown. The structures were predicted by AlphaFold methods, except the crystal structure of *C. elegans* PRMT5 (PDB code 3UA3).

Supplementary Fig. 4

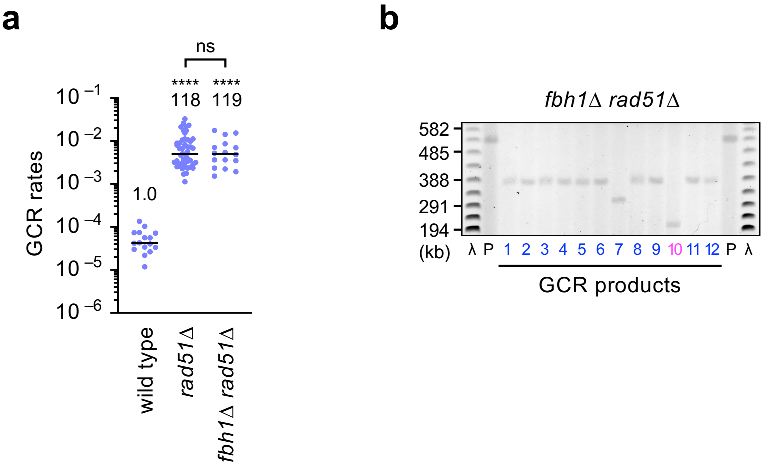

Supplementary Fig. 4 Fbh1 is dispensable for the isochromosome formation in *rad51*Δ cells. a GCR rates of wild-type, *rad51*Δ*,* and *fbh1*Δ *rad51*Δ strains (TNF5369, 5411, and 8826). The two-tailed Mann-Whitney test. **b** GCR products of the *fbh1*Δ *rad51*Δ strain were separated by PFGE. Sample numbers of isochromosomes and truncations are shown in blue and magenta, respectively. Uncropped gel images are shown in Supplementary Fig. 5.

Supplementary Fig. 5

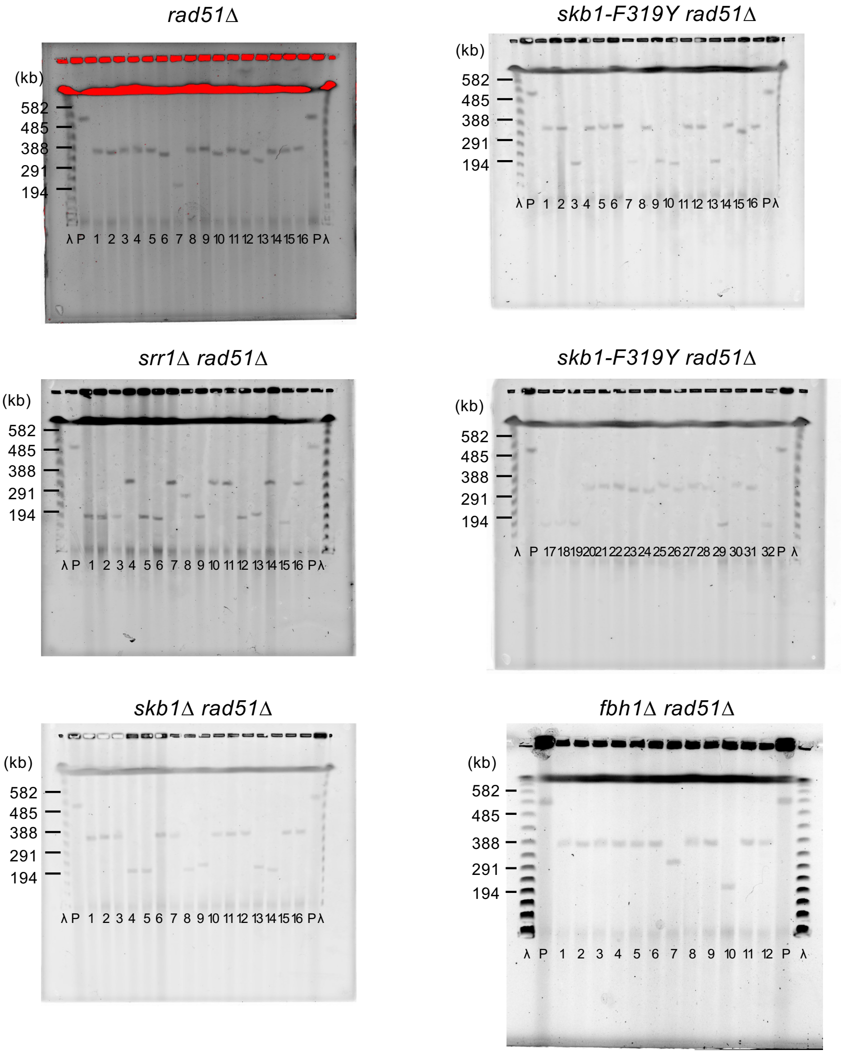

Supplementary Fig. 5 Uncropped images of gels in Figs. 2b, 6d, and Supplementary Fig. 4.

Supplementary Fig. 6

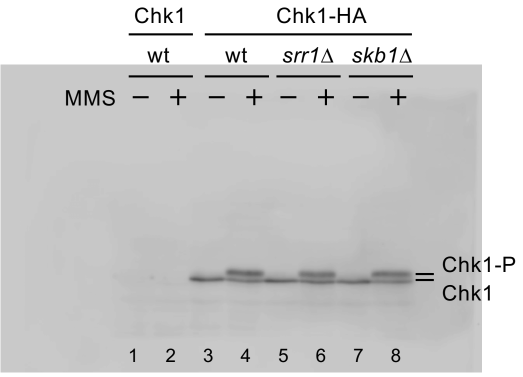

anti-HA immunostaining

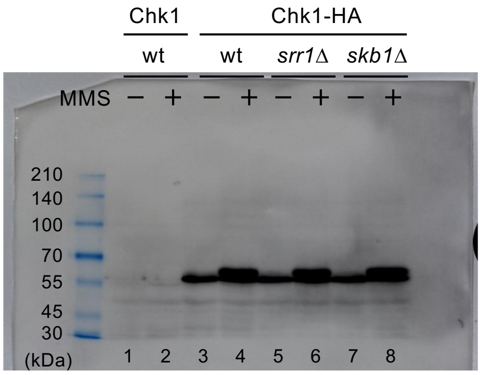

anti-HA immunostaining and
CLEARLY stained protein ladder
overlayed image automatically generated by ImageQuant LAS 500

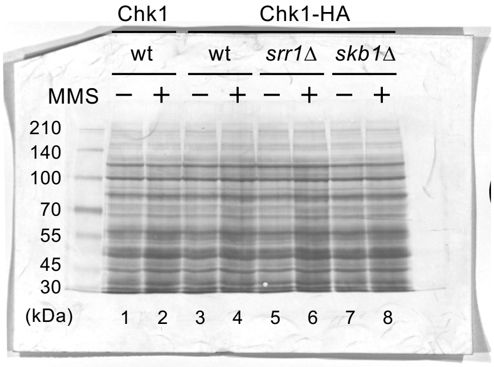

Coomassie brilliant blue staining

Supplementary Fig. 6 Uncropped images shown in Fig. 3c. CLEARLY stained protein ladder (Takara, 3454A) was used as size markers.

Supplementary Table 1

Supplementary Table 1. Yeast strains used in this study.	
Strain	Genotype
TNF5369	*h- (smt0), leu1-32, ura4-D18, ade6Δ-D, ChLC (ubc11::LEU2+, cwf20::ura4+, ade6+)*
TNF5411	*h- (smt0), leu1-32, ura4-D18, ade6Δ-D, ChLC (ubc11::LEU2+, cwf20::ura4+, ade6+), rad51::natMX6*
TNF5954	*h- (smt0), leu1-32, ura4-D18, ade6Δ-D, ChLC (ubc11::LEU2+, cwf20::ura4+, ade6+), srr1-W157R, skb1-A377V, rad51::natMX6* (a randomly mutagenized clone of TNF5411)
TNF5772	*h- (smt0), leu1-32, ura4-D18, ade6Δ-D, ChLC (ubc11::LEU2+, cwf20::ura4+, ade6+), skb1::kanM6X*
TNF5774	*h- (smt0), leu1-32, ura4-D18, ade6Δ-D, ChLC (ubc11::LEU2+, cwf20::ura4+, ade6+), srr1::kanMX6*
TNF5904	*h- (smt0), leu1-32, ura4-D18, ade6Δ-D, ChLC (ubc11::LEU2+, cwf20::ura4+, ade6+), srr1::kanMX6, rad51::natMX6*
TNF5788	*h- (smt0), leu1-32, ura4-D18, ade6Δ-D, ChLC (ubc11::LEU2+, cwf20::ura4+, ade6+), skb1::kanMX6, rad51::natMX6*
TNF8432	*h- (smt0), leu1-32, ura4-D18, ade6Δ-D, ChLC (ubc11::LEU2+, cwf20::ura4+, ade6+), srr1::hphMX6, skb1::kanMX6, rad51::natMX6*
TNF8391	*h- (smt0), leu1-32, ura4-D18, ade6Δ-D, ChLC (ubc11::LEU2+, cwf20::ura4+, ade6+), skb1-F319Y, rad51::natMX6*
TNF8474	*h- (smt0), leu1-32, ura4-D18, ade6Δ-D, ChLC (ubc11::LEU2+, cwf20::ura4+, ade6+), skb1-E422A,E431A, rad51::natMX6*
TNF8359	*h- (smt0), leu1-32, ura4-D18, ade6Δ-D, ChLC (ubc11::LEU2+, cwf20::ura4+, ade6+), skb1-A377V, rad51::natMX6*
TNF8344	*h- (smt0), leu1-32, ura4-D18, ade6Δ-D, ChLC (ubc11::LEU2+, cwf20::ura4+, ade6+), srr1-W157R, rad51::natMX6*
TNF8547	*h- (smt0), leu1-32, ura4-D18, ade6Δ-D, ChLC (ubc11::LEU2+, cwf20::ura4+, ade6+), srr1-W157R, skb1-A377V, rad51::natMX6*
TNF8308	*h- (smt0), leu1-32, ura4-D18, ade6Δ-D, ChLC (ubc11::LEU2+, cwf20::ura4+, ade6+), srr1-W157R*
TNF6599	*h- (smt0), leu1-32, ura4-D18, ade6Δ-D, ChLC (ubc11::LEU2+, cwf20::ura4+, ade6+), rad52-R45K*
TNF7122	*h- (smt0), leu1-32, ura4-D18, ade6Δ-D, ChLC (ubc11::LEU2+, cwf20::ura4+, ade6+), rad52-R45K, rad51::natMX6*
TNF8663	*h- (smt0), leu1-32, ura4-D18, ade6Δ-D, ChLC (ubc11::LEU2+, cwf20::ura4+, ade6+), srr1-W157R, rad52-R45K, rad51::natMX6*
TNF8281	*h- (smt0), leu1-32, ura4-D18, ade6Δ-D, ChLC (ubc11::LEU2+, cwf20::ura4+, ade6+), srr1::kanMX6, rad52-R45K*
TNF8324	*h- (smt0), leu1-32, ura4-D18, ade6Δ-D, ChLC (ubc11::LEU2+, cwf20::ura4+, ade6+), skb1::kanMX6, rad52-R45K*
TNF8345	*h- (smt0), leu1-32, ura4-D18, ade6Δ-D, ChLC (ubc11::LEU2+, cwf20::ura4+, ade6+), skb1::kanMX6, rad52-R45K, rad51::natMX6*
TNF6761	*h- (smt0), ade6Δ-D, leu1-32, ura4-D18, ChLC (ubc11::LEU2+, cwf20::ura4+, ade6+), pcn1-K107R, rad51::natMX6*
TNF8601	*h- (smt0), ade6Δ-D, leu1-32, ura4-D18, ChLC (ubc11::LEU2+, cwf20::ura4+, ade6+), srr1-W157R, pcn1-K107R, rad51::natMX6*
TNF8387	*h- (smt0), leu1-32, ura4-D18, ade6Δ-D, ChLC (ubc11::LEU2+, cwf20::ura4+, ade6+), srr1-H148A, rad51::natMX6*
TNF8686	*h- (smt0), leu1-32, ura4-D18, ade6Δ-D, ChLC (ubc11::LEU2+, cwf20::ura4+, ade6+), srr1-D111A,P112A, rad51::natMX6*
TNF8826	*h- (smt0), leu1-32, ura4-D18, ade6Δ-D, ChLC (ubc11::LEU2+, cwf20::ura4+, ade6+), fbh1::kanMX6 , rad51::natMX6*
TNF8811	*h- (smt0), leu1-32, ura4-D18, ade6Δ-D, ChLC (ubc11::LEU2+, cwf20::ura4+, ade6+), slf1::hphMX6*
TNF8813	*h- (smt0), leu1-32, ura4-D18, ade6Δ-D, ChLC (ubc11::LEU2+, cwf20::ura4+, ade6+), pom1::hphMX6*
TNF8834	*h- (smt0), leu1-32, ura4-D18, ade6Δ-D, ChLC (ubc11::LEU2+, cwf20::ura4+, ade6+), slf1::hphMX6 , rad51::natMX6*
TNF8838	*h- (smt0), leu1-32, ura4-D18, ade6Δ-D, ChLC (ubc11::LEU2+, cwf20::ura4+, ade6+), pom1::hphMX6 , rad51::natMX6*
TNF35	*h+*
TNF5943	*h+, srr1::kanMX6*
TNF9011	*h+, srr1-W157R*
TNF9007	*h+, srr1-H148A*
TNF8990	*h+, srr1-D111A,P112A*
TNF8107	*h+, rad51::natMX6*
TNF8188	*h+, srr1::kanMX6, rad51::natMX6*
TNF8321	*h+, skb1::kanMX6*
TNF3559	*h+, chk1::kanMX6*
TNF7555	*h- (smt0), mat2-3::natMX6*
TNF8441	*h- (smt0), mat2-3::natMX6, chk1-6His3HA:kanMX6*
TNF8799	*h- (smt0), mat2-3::natMX6, chk1-6His3HA:kanMX6, srr1::hphMX6*
TNF8802	*h- (smt0), mat2-3::natMX6, chk1-6His3HA:kanMX6, skb1::hphMX6*
TNF8320	*h+, skb1::kanMX6, rad51::natMX6*
TNF7988	*h- (smt0), leu1-32, rad52::hphMX6*
TNF3885	*h- (smt0), leu1-32, ura4-D18, ade6Δ-D*
TNF8573	*h- (smt0), leu1-32, ura4-D18, ade6Δ-D, srr1-W157R, rad51::natMX6*
TNF5845	*h- (smt0), leu1-32, ura4-D18, ade6Δ-D, rad51::natMX6*
TNF5847	*h- (smt0), leu1-32, ura4-D18, ade6Δ-D, srr1::kanMX6*
TNF5849	*h- (smt0), leu1-32, ura4-D18, ade6Δ-D, srr1::kanMX6, rad51::natMX6*
TNF8280	*h- (smt0), leu1-32, ura4-D18, ade6Δ-D, srr1-W157R*
TNF8275	*h- (smt0), leu1-32, ura4-D18, ade6Δ-D, srr1-H148A*
TNF8274	*h- (smt0), leu1-32, ura4-D18, ade6Δ-D, srr1-D111A,P112A*
TNF8614	*h+, rad52-AID-Turg1:kanMX6*
TNF8621	*h+, rad52-AID-Turg1:kanMX6, srr1::hphMX6*
TNF8616	*h+, arg3::bleMX6-arg3+-Padh1-OsTIR1F74A-TADH1*
TNF8623	*h+, arg3::bleMX6-arg3+-Padh1-OsTIR1F74A-TADH1, srr1::hphMX6*
TNF8617	*h+, arg3::bleMX6-arg3+-Padh1-OsTIR1F74A-TADH1, rad52-AID-Turg1:kanMX6*
TNF8627	*h+, arg3::bleMX6-arg3+-Padh1-OsTIR1F74A-TADH1, rad52-AID-Turg1:kanMX6, srr1::hphMX6*
TNF5492	*h+, rpa2-mCherry:hphMX6*
TNF8803	*h+, rpa2-mCherry:hphMX6, srr1::kanMX6*
TNF4442	*h+, rad52-GFP:hphMX6*
TNF6130	*h+, rad52-GFP:hphMX6, srr1::kanMX6*

Supplementary Table 2

Supplementary Table 2. Oligonucleotide sequences		
stock #	oligo name	sequence
2398	srr1-F1	5'-TCGCTGCAATTGGAACCGGG
2399	srr1-R1	5'-GGGCAAGCGGTATTTCGTAATCGC
2400	srr1-ura4AN5	5'-GCCAGTGGGATTTGTAGCTATAGTGAAGTAGGGCAGTGTGGC
2401	srr1-ura4AN3	5'-GGCGTTTTATGTCAGAAGGCGCCTATGTAAATGATGATCCCAGGC
1741	srr1-kan5	5'-CCCGGCGGGGACGAGGCTAAAGTGCAGGAAGGCCCAACAC
1742	srr1-kan3	5'-CGATACTAACGCCGCCATCCAGGCATTTTATCATGTGCGGCAAC
1740	srr1-1	5'-TCCGTGCAGAAATCGCTACATTC
1744	srr1-3	5'-GGGCAAGCGGTATTTCGTAATCG
1737	skb1-kan3	5'-CGATACTAACGCCGCCATCCGGTTTAAAACGGGTCTTCAAACC
1736	skb1-kan5	5'-CCCGGCGGGGACGAGGCGTAAGGGTTGGGACCTTGTGTTGC
1735	skb1-1	5'-TCCATTACAAAGCGGTGGAAGTG
1738	skb1-2	5'-GTGCCAAATACGGGTTCTTTGAC
1739	skb1-3	5'-AGCTGCTTGTGCAACAGTCG
2412	skb1-F1	5'-GGCCTTAGAGCTACCTCCCGC
2413	skb1-R1	5'-GACTTCGCTTCCATAGTTGCTGGC
2414	skb1-ura4AN5	5'-GCCAGTGGGATTTGTAGCTACCGTTCTAGATGAAGAAATTGCCGC
2415	skb1-ura4AN3	5'-GGCGTTTTATGTCAGAAGGCCAGGATTGGGCTGGCAAAG
2418	skb1-F319Y-F	5'-CATTACTTATGAAATATATGAGCGAGATCCCGTTAAG
2419	skb1-F319Y-R	5'-CTTAACGGGATCTCGCTCATATATTTCATAAGTAATG
2465	skb1-doubleE-F	5'-GTGCACTTTTGGGTAGTATGGGAGACAATGCACTTAGTCCGGAGTGTC
2466	skb1-doubleE-R	5'-CTAAGTGCATTGTCTCCCATACTACCCAAAAGTGCACTAACAAGGATGTC
1519	rad52-N-F2	5'-GCCAGTGGGATTTGTAGCTAAGAATTGGGTTATTTTCATGTTTGG
1525	rad52-C-R	5'-CCTCTGCTACTGCTAAATGAGC
2422	srr1-H148A-F	5'-CACTCTACTTTATATGCCGGCCTGCCCTACTTCACTATATGAAACTTGG
2423	srr1-H148A-R	5'-CCAAGTTTCATATAGTGAAGTAGGGCAGGCCGGCATATAAAGTAGAGTG
2420	srr1-DPAA-F	5'-GATTTTGTTCATTTTATGCTGCAGCATTTCTCAAAGATGATGTCG
2421	srr1-DPAA-R	5'-CGACATCATCTTTGAGAAATGCTGCAGCATAAAATGAACAAAATC
2616	slf1-F1	5'-CCATCACACTTCACAGTGTTGATGG
2617	slf1-R-K5	5'-CCCGGCGGGGACGAGGCTCATTCAAGTAAATACACGCAAACTG
2618	slf1-F-K3	5'-CGATACTAACGCCGCCATCCGTCGTTTATCTTCCTTGCTACTAAGC
2619	slf1-R1	5'-GGGTACTTACGACCATAAAGTTGTCG
2620	slf1-R2	5'-GATTACTACCGCGCTCGCTGT
2611	pom1-F1	5'-CAGCGAGCAATTTAATTTGTTCTGC
2612	pom1-R-K5	5'-CCCGGCGGGGACGAGGCATTTGCAAACTTTGCTTCAAAAAGCC
2613	pom1-F-K3	5'-CGATACTAACGCCGCCATCCGTGATCACACATTACCTTGGAATAGG
2614	pom1-R1	5'-GCACACTAACTTGAATACTCGTGTCC
2615	pom1-R2	5'-TGGTCCAAAGGAGCAACCCTGG
2408	fbh1-F0	5'-ACCAACCAAGAGGTCGTTGATGAG
2404	fbh1-F1	5'-ATTGGAATTTGCCAAGTCTCGTCC
2405	fbh1-F2	5'-ACAATCGATTCGGCTATCAGTGTC
2410	fbh1-F3	5'-GTAGGACTACTACTAGCAATCTGC
2411	fbh1-F4	5'-TGTCATCGTTGGGGATGCACACC
2409	fbh1-R0	5'-ACACGAAGGGTTATTTCCATGGC
2406	fbh1-R1	5'-CGCTCAAGTTTAGCTTGCGGC
2407	fbh1-R2	5'-GCACTTCAAATTCTCCCCAAGAGG

Nucleotides underlined are mutation sites.
