# Supplementary material for: Fission yeast Srr1 and Skb1 promote isochromosome formation at the centromere
Source: Commun Biol. 2023 May 26;6:551. doi: 10.1038/s42003-023-04925-9 (PMC10219947; doi:10.1038/s42003-023-04925-9)
Supplement: Supplementary file 3 — Description of Additional Supplementary Files [file 42003_2023_4925_MOESM3_ESM.pdf]

Description of Additional Supplementary Files

File name: Supplementary Data 1 (Excel file)

Description: The source data behind the graphs.

File name: Supplementary Data 2 (Excel file)

Description: A list of proteins that show 3D similarity to the SRR1-like domain.

File name: Supplementary Data 3 (Zip file)

Description: DNA sequencing data of the fbh1 gene (SnapGene file).
