# Supplementary material for: Fission yeast Srr1 and Skb1 promote isochromosome formation at the centromere
Source: Commun Biol. 2023 May 26;6:551. doi: 10.1038/s42003-023-04925-9 (PMC10219947; doi:10.1038/s42003-023-04925-9)
Supplement: Supplementary file 7 — Reporting Summary [file 42003_2023_4925_MOESM7_ESM.pdf]

nature portfolio

Corresponding author(s): Takuro Nakagawa

Last updated by author(s): May 3, 2023

Reporting Summary

Nature Portfolio wishes to improve the reproducibility of the work that we publish. This form provides structure for consistency and transparency in reporting. For further information on Nature Portfolio policies, see our Editorial Policies and the Editorial Policy Checklist.

Statistics

For all statistical analyses, confirm that the following items are present in the figure legend, table legend, main text, or Methods section.

n/a	Confirmed	
☐	☒	The exact sample size (*n*) for each experimental group/condition, given as a discrete number and unit of measurement
☐	☒	A statement on whether measurements were taken from distinct samples or whether the same sample was measured repeatedly
☐	☒	The statistical test(s) used AND whether they are one- or two-sided<br>*Only common tests should be described solely by name; describe more complex techniques in the Methods section.*
☒	☐	A description of all covariates tested
☒	☐	A description of any assumptions or corrections, such as tests of normality and adjustment for multiple comparisons
☐	☒	A full description of the statistical parameters including central tendency (e.g. means) or other basic estimates (e.g. regression coefficient) AND variation (e.g. standard deviation) or associated estimates of uncertainty (e.g. confidence intervals)
☐	☒	For null hypothesis testing, the test statistic (e.g. *F*, *t*, *r*) with confidence intervals, effect sizes, degrees of freedom and *P* value noted<br>*Give P values as exact values whenever suitable.*
☒	☐	For Bayesian analysis, information on the choice of priors and Markov chain Monte Carlo settings
☒	☐	For hierarchical and complex designs, identification of the appropriate level for tests and full reporting of outcomes
☒	☐	Estimates of effect sizes (e.g. Cohen's *d*, Pearson's *r*), indicating how they were calculated

Our web collection on statistics for biologists contains articles on many of the points above.

Software and code

Policy information about availability of computer code

Data collection	Microsoft Excel for Mac 16.72 was used to collect the number of yeast colonies when we measured the rate of GCR and chromosome loss.
Data analysis	The two-tailed Mann-Whitney and the two-tailed Fischer's exact tests were performed using GraphPad Prism 9 for MacOS. The two-tailed student's t-test and Pearson's Chi-square test were performed using Microsoft Excel for Mac 16.72. Gel images were processed using ImageJ2 2.9.0 or Adobe Photoshop elements 2020 software.

For manuscripts utilizing custom algorithms or software that are central to the research but not yet described in published literature, software must be made available to editors and reviewers. We strongly encourage code deposition in a community repository (e.g. GitHub). See the Nature Portfolio guidelines for submitting code & software for further information.

Data

Policy information about availability of data

All manuscripts must include a data availability statement. This statement should provide the following information, where applicable:

- Accession codes, unique identifiers, or web links for publicly available datasets
- A description of any restrictions on data availability
- For clinical datasets or third party data, please ensure that the statement adheres to our policy

The data supporting the findings of this study are included in the paper and the supplementary information. All raw datasets are available from the corresponding author upon reasonable request.

Human research participants

Policy information about studies involving human research participants and Sex and Gender in Research.

Reporting on sex and gender	N/A
Population characteristics	N/A
Recruitment	N/A
Ethics oversight	N/A

Note that full information on the approval of the study protocol must also be provided in the manuscript.

Field-specific reporting

Please select the one below that is the best fit for your research. If you are not sure, read the appropriate sections before making your selection.

[x] Life sciences [ ] Behavioural & social sciences [ ] Ecological, evolutionary & environmental sciences

For a reference copy of the document with all sections, see nature.com/documents/nr-reporting-summary-flat.pdf

Life sciences study design

All studies must disclose on these points even when the disclosure is negative.

Sample size	At least 16 and 5 biologically independent experiments were performed for each strain by taking independent colonies, when we determined the rates of GCR and chromosome loss, respectively. We counted >300 nuclei each to detect the Rpa2-mCherry and Rad52-GFP focus, respectively, in 3 independent cultures. We also counted >300 cells to detect septa in each strain. We analyzed at least 12 GCR products by PFGE in each strain tested.
Data exclusions	No data were excluded from the analyses.
Replication	All attempts at replication were successful.
Randomization	In the GCR assay, we recovered both large and small colonies for PFGE, according to the ratio of their appearance.
Blinding	No blind experiments.

Reporting for specific materials, systems and methods

We require information from authors about some types of materials, experimental systems and methods used in many studies. Here, indicate whether each material, system or method listed is relevant to your study. If you are not sure if a list item applies to your research, read the appropriate section before selecting a response.

Materials & experimental systems

n/a	Involved in the study	
[ ]	[x]	Antibodies
[x]	[ ]	Eukaryotic cell lines
[x]	[ ]	Palaeontology and archaeology
[x]	[ ]	Animals and other organisms
[x]	[ ]	Clinical data
[x]	[ ]	Dual use research of concern

Methods

n/a	Involved in the study	
[x]	[ ]	ChIP-seq
[x]	[ ]	Flow cytometry
[x]	[ ]	MRI-based neuroimaging

Antibodies

Antibodies used	Anti-HA antibody (16B12, Abcam, Cambridge, MA). Peroxidase AffiniPure goat anti-mouse IgG (heavy + light) (Jackson ImmunoResearch Laboratories, 115-035-146).
Validation	We detected an immunostaining Chk1-HA band when we used the extract prepared from the cells expressing Chk1-HA but not from the cells expressing Chk1.
